# Omnipresence, Victim-Shaming and Non-Action: Young People's Views on Sexual Harassment in the #Metoo Era

**DOI:** 10.1177/10778012251329176

**Published:** 2025-03-19

**Authors:** Satu Venäläinen

**Affiliations:** 1 Helsinki Collegium for Advanced Studies, 3835 University of Helsinki, Helsinki, Finland

**Keywords:** #metoo, sexual harassment, young people, understandings/experiences

## Abstract

This study explores the views and experiences of sexual harassment among youth and young adults aged 15–29 years in the wake of the #metoo movement in Finland. The data were collected through a qualitative survey and interviews and analyzed using reflexive thematic analysis. The study contributes to the literature that foregrounds young people's own voices by shedding light on the participants’ experience-based knowledge of sexual harassment. The analysis generated three predominant themes: the insidious omnipresence of sexual harassment, the persistence of victim-shaming, and non-action as the prevalent norm. Together, these themes highlight the participants’ readiness to engage in a critique of sexual harassment while simultaneously showing its intense and continuing salience and harmfulness in their everyday lives.

Since the #metoo movement, a frequently asked question has been whether any change has occurred regarding awareness and the practices that sustain sexual harassment. A large body of research (see e.g., Fileborn & Loney-Howes, 2019; Quan-Haase et al., 2021) focused particularly on social media has considered the movement's role in increasing public awareness of sexual harassment and violence, and their underpinnings in gendered inequalities. While several studies (e.g., Kilimnik et al., 2024; Mendes et al., 2018a) have emphasized the movement's potential to strengthen the possibilities to name one's experiences as harassment and to criticize the gendered inequalities to which sexual harassment is linked, this optimism has been accompanied by caution regarding the movement's capacity to induce concrete and widespread change. In particular, online manifestations of the #metoo movement have been associated with neoliberal feminism, which tends to individualize problems such as gender-based violence instead of constituting a politically effective, collective effort to expose and alter social injustice (Ashworth & Pedersen, 2023; Horeck et al., 2023; Rottenberg, 2019). Similarly, #metoo has been seen to reproduce existing intersectional inequalities by foregrounding the voices of already privileged groups of women and girls, while marginalizing racialized women's voices, as well as those of gender or sexual minorities (Mendes et al., 2018a; Trott, 2020). Therefore, the potentially empowering effect of the #metoo movement might be limited: On a positive note, the understandings of sexual harassment that the #metoo movement has cultivated and spread are continuous with earlier decades of feminist activism and gender-sensitive research on sexual violence, which enable seeing sexual harassment and violence as a systemic issue based on and reproducing gendered and intersectional inequalities. However, due to their affinities with popular, neoliberal forms of feminism, social media movements such as #metoo may highlight individualistic accounts of sexual harassment and violence experiences that obscure the societal-level influences on them, and thus easily dilute political and intersectional engagement with the issue (Boyle, 2024; Horeck et al., 2023; Rottenberg, 2018, 2019).

Young people have played a key role in the spread of social media movements that challenge sexual harassment and are frequently considered particularly adept at criticizing gendered and intersectional inequalities (Jackson, 2018; Mendes et al., 2018b; Sills et al., 2016). However, despite increased critique against sexual harassment, many young people continue to be subjected to harassment and violence in their everyday lives, both offline and online (Skoog et al., 2023). This creates a dilemma of increased opportunities to adopt a critical, empowered stance against sexual harassment and violence, on the one hand, and the continued prevalence of sexual harassment and its negative effects on young people's well-being, on the other (Venäläinen & Calder-Dawe, 2024). To understand how young people perceive and manage this dilemma, it is important to analyze their own sense-making regarding both sexual harassment and the current possibilities to intervene in it.

Overall, studies that aim to highlight young people's voices and their own interpretations of sexual harassment and violence have begun to increase in recent years (Mendes et al., 2018b; Pihkala et al., 2019; Renold, 2018). Studies (e.g., Honkatukia et al., 2023; Horeck et al., 2023) with a particular focus on young people's awareness of sexual harassment and their ability to relate to it as a systemic issue in the wake of the #metoo movement have indicated that many do indeed make sense of sexual harassment and violence with an emphasis on gendered and societal power imbalances. However, there is also evidence of gender differences in the extent to which such understandings have been adopted. While women's and girls’ understandings of the issue frequently highlight its severity and link it with gender inequalities, men and boys appear to downplay its significance more often and might even align with antifeminist views that disregard the gendered nature of the issue (Honkatukia et al., 2023; Horeck et al., 2023).

In sum, existing research suggests that young people's views on sexual harassment might indeed have shifted in recent years, but these shifts may inform women and girls’ understandings to a greater extent than men's and boys’. Nonbinary young people's views are severely understudied. Furthermore, the potential spread of critical views continues to clash with the reality of continuing prevalence of both sexual harassment and antifeminist discourses in young people's everyday lives. This complexity calls for analyses that pay close attention to young people's own understandings of the problem, and also highlight their proposed solutions in intervention efforts (Ringrose et al., 2025). This paper answers to this call by exploring views on sexual harassment and the best ways to intervene in it among 15- to 29-year-old women, men, and nonbinary young people living in Finland. Due to the wide age range, the participants include both youth and young adults, but in accordance with the Finnish legal definition of youth, the participants are considered here as representatives of a same broad group labeled as young people.

The analysis shows that these young people's ways of relating to sexual harassment are characterized by a strong alignment with a critical stance that emphasizes the insidious omnipresence of sexual harassment, the persistence of victim-shaming, and the prevalence of non-action. The analysis thereby suggests that sexual harassment is understood and experienced by young people in many ways as both an overwhelming and a solvable problem. A secondary aim of the analysis is to highlight the intertwining of young people's understandings and their personal experiences of harassment and, in doing so, to give specific value to their experience-based knowledge of harassment and the best ways to tackle it. This interpretive lens is built on the theorization of situated knowledges as well as a new materialist understanding of onto-epistemologies, which will be discussed in more detail in the next section.

## The Gendered Contours of Sexual Harassment and Its Understandings/Experiences

Sexual harassment is commonly defined as a range of behaviors that are experienced as violating a person's right to self-determination. Feminist theory places sexual harassment in a broader continuum of sexual and gender-based violence, and thus views it as intimately linked with gendered inequalities (Kelly, 2012). Studies on sexual harassment among young people have illuminated its function as a means to reproduce gendered hierarchies: by harassing and thereby objectifying girls, boys and men enact their distinctiveness and empowered position in relation to them (Aaltonen, 2017; Renold, 2002; Robinson, 2005). Similarly, when targeting gender and/or sexual minority young people, sexual and gender-based harassment are means to maintain the status quo aligned with gender and sexual norms by subjugating those who are seen as not conforming to them.

Previous studies (e.g., Ringrose et al., 2021; Virrankari & Leemann, 2022) conducted both in Finland and in other Westernized countries, such as the United Kingdom, have shown that experiencing sexual harassment is not only frequent but can also have serious social and psychological consequences. In studies focused on young people, such experiences have been found to cause feelings of insecurity and fear, among other aftereffects, and to correlate with declines in academic performance and social relationships in school (Gruber & Fineran, 2016). Young people who have experienced sexual harassment have also been found to have a lower sense of social inclusion. These consequences can affect the willingness of victims to report harassment they encounter and seek help and support (Virrankari & Leemann, 2022).

Despite the emotional and social harm that sexual harassment causes, the meanings given to harassment and the ways of making sense of it are not stable, nor is the harm it causes necessarily recognized. Rather, its definitions and the boundaries between sexual harassment and non-harassing interaction can vary over time and place, and accordingly, the ways of responding to it in everyday encounters vary (Aaltonen, 2017). Corresponding shifts also occur in the public imagery and the underlying discourses related to sexual harassment, which in recent years have evolved closely in sync with social media movements such as the #metoo (Lazard, 2020; Venäläinen et al., 2023). Because of this variability, it is not self-evident which experiences are named as harassment, how they are experienced, and what kind of solutions are prioritized. What matters in youth's and young adult's ways of experiencing and viewing sexual harassment are not only publicly circulating discourses but also the ways these relate to their everyday encounters with people, institutions, and material and virtual (including offline and online) spaces.

The analysis presented in this paper is based first on insights from previous research reviewed above, which has viewed sexual harassment as a violent practice that enacts gendered inequalities. Harassment is therefore seen as gaining force from normative understandings of gender, which work to normalize sexual harassment and violence and thereby obscure its violent and gendered nature (Gavey, 2019). Second, this understanding is combined in the current analysis with the notion of situated knowledges (Haraway, 1998) and a new materialist understanding of onto-epistemology (e.g., Davies, 2020). While the notion of situated knowledges underscores the nature of all knowledge as partial and experience-based, new materialist perspectives underscore both the entangled and the constantly evolving nature of ontology and epistemology. Thus, the analysis is based on the view that discursive sense-making is inseparable from the material manifestations of sexual harassment and violence, including their effects on the victims and societal power relations at large (Venäläinen, 2023). This has two main analytical implications. First, the analysis is based on the assumption that shifts in discourses, such as the spread of condemnation of sexual harassment in the wake of the #metoo movement, are linked with potential shifts in material practices, while also recognizing that the nature and scope of such shifts can be multifold and unpredictable. Second, the analysis aims to illuminate the lived experiences of the research participants, which, however, are seen as mediated in their nature. The idea of mediation here refers to viewing any discursively accomplished account of an experience as shaped by the discursive means, or affordances, that enable making the experience intelligible. Thus, the ways ontology and epistemology intertwine are seen here as multilayered and as multiply-inscribed into the research materials and their analysis. To underscore this, the analysis identifies what I label *understandings/experiences*—fusions of everyday experiences and the discursive patterns that shape both the experiences and their telling.

## Materials and Methods

The materials analyzed in this paper were collected in Finland, where, similar to other Nordic and more broadly Western country contexts, high rates of sexual harassment among young people have been observed in recent surveys. A nationwide school health promotion study indicated that approximately 50% of girls and 8% of boys (15- to 16-year-olds) reported experiencing sexual harassment (Helakorpi & Kivimäki, 2021). The data for the current study consist of online interviews and qualitative online survey responses, which were collected during roughly the same period as the nationwide quantitative survey—between January 2021 and June 2022. The data were collected for a larger project which included organizing collaborative workshops for young people. Even though the workshops are not analyzed as “data” in this paper, as I discuss in the concluding section of the paper, they can be considered as having provided background knowledge, which helped confirm some of the interpretations made on the basis of the online interviews and qualitative survey responses. Participants were recruited with the help of various NGOs (non-governmental organizations) that provide services for young people and municipal youth services in four towns in southern Finland. Each organization granted a research permit before the collection of materials, and they mostly used their social media channels to reach out to potential participants. Many NGOs that assisted in recruiting participants are focused on providing networks and peer support for sexual- and gender-minority youth. This enabled reaching out to LGBTIQ+ young people whose voices have frequently been omitted from research on sexual harassment despite the heightened risks they face (McKay et al., 2019).

The qualitative survey and the interviews included largely similar questions, mainly focused on (1) perceptions of sexual harassment's prevalence and its forms among young people; (2) the places in which sexual harassment occurs; (3) whether the participant had first-hand experiences of harassment to share; (4) their feelings about sexual harassment; and (5) their thoughts on social media discussions around sexual harassment, (6) how sexual harassment could be prevented and (7) how the victims/survivors of sexual harassment could be helped. The online form also provided a space for the participants to write freely about the topic. The interview questions were largely the same as the questions on the internet form, but also included additional questions that focused on how the participants define sexual harassment, and their views on the significance of gender and sexual orientation in sexual harassment and on its origins and consequences. The online survey, in turn, defined sexual harassment for participants as “unwanted sexual behavior directed at another person and/or occurring without the person's consent.” After participating in the survey or interview, participants were offered a list of services providing help and assistance to young people (including digital services).

A total of 105 participants aged 15–29 years provided written responses to the qualitative survey. Most participants were under the age of 20 (*Mdn*: 16). In addition, 11 participants ranging in age from 15 to 29 years (*Mdn*: 24) took part in an online interview (conducted via Zoom). Of all the participants, the majority (49 participants), were in their middle teens (15–17 years old), nine were in their late teens (18–20 years old), 20 were in their early twenties (21–25 years old) and 13 were in their late twenties (26–29 years old). Although those who participated in the interviews were older on average than the survey respondents, their views and experiences typically aligned closely with those of the younger participants. The participants were given the choice of participating in the interviews in Finnish or in English. Six interviews were conducted in Finnish and five in English. Those who participated in the English language had transnational backgrounds, and the experiences and observations they shared were thus often not limited to Finland. Rather than differences, these participants’ experiences highlighted continuities across cultural contexts and backgrounds. A clear majority of participants had lived most of their lives in Finland. The duration of the interviews ranged from 38 min to 2 hr and 40 min (*Mdn*: 1 hr 20 min).

The majority of participants (73) self-identified as women, 25 self-identified as men, and 18 self-identified in nonbinary or non-conforming terms (six identified as nonbinary, one as gender fluid, one as gender apathetic, one as agender, five as questioning and four did not define their gender). The participants were given the option to self-define their gender identity or not, as they wished. Other demographic information requested was likewise optional, based on the participants’ preferences of giving only information they wanted to. A total of 43 participants reported belonging to gender and/or sexual minorities, including the gender-minority identities listed above (*n *= 18) and transgender identities (*n *= 2), and/or identifying as queer, bisexual, asexual, pan-, demi-, or omni-sexual. To protect the participants’ anonymity, only their pseudonyms (chosen by the author), age, and self-defined gender identity are given in the analysis section in connection with the extracts. Informed consent was obtained from all participants, and it was stressed that participation in the study was voluntary. The interviews were audiotaped with the participants’ permission and transcribed verbatim. The materials were anonymized at the point of transcription. The materials were analyzed in their original languages; extracts that were originally in Finnish were translated by the author for this paper.

The analysis followed the principles of reflexive thematic analysis (RTA) (Braun & Clarke, 2012), which is characterized by the importance given to reflexivity as well as flexibility regarding the theorization combined with the analysis. This suited the present study's objective of highlighting young people's voices and experience-based knowledge. The formulation of these objectives was, in turn, informed by a reflexive consideration of researchers’ positionality in relation to the participants. Because the research was conducted by a ciswoman researcher, not a young person herself, with previous experience in feminist research on gender-based violence, it was deemed important to listen to young people's own understandings as closely as possible, and to generate interpretations that highlight the grounding of their understandings in their lived experiences. In practice, moving through the analytical phases included in RTA, from initial familiarization with the materials and initial coding through the refinement and final naming of the themes generated, afforded the analyst an increasingly in-depth engagement with the materials. This yielded three themes that, while not exhausting the analytical potential and nuances in the materials, crystallized the patterns of the meanings stressed most poignantly by the participants. The themes were, in short, the insidious omnipresence of sexual harassment, the persistence of victim-shaming, and non-action as the prevalent norm, each of which is presented and discussed in more detail below.

## Analysis

### The Insidious Omnipresence of Sexual Harassment

The first understanding/experience that united most participants’ accounts highlighted the seriousness of sexual harassment as an issue, often through recounting the participants’ own frequent encounters with it. The participants stressed rather univocally the frequency of occurrences of sexual harassment among young people, and thus saw it as an omnipresent part of their everyday lives. They repeatedly made the point that sexual harassment happens “everywhere,” not limited to particular, risky spaces or events that could be avoided, but rather is a constant risk in their lives: “In school, on the streets, in the shop, everywhere, it has become a social norm” (girl, age 15, written account). The notion of “everywhere” included online spaces, which the participants mostly viewed as similar to offline spaces in terms of risk, somewhat deviating from previous findings that young people reported the risk of harassment in online spaces as easier to manage than in offline spaces (Honkatukia et al., 2023). However, while this *everywhereness* of sexual harassment was widely agreed upon, it had an insidious nature: sexual harassment and the harm it caused appeared both as tangible and simultaneously so routine that it appeared submerged in the participants’ everyday experiences. As the extracts in this section illustrate, this was also bound with a complex relationship that formed between experiencing and naming embodied violations.

The severity of sexual harassment highlighted by many participants was often bound with their recollections of their own experiences of harassment. The following account by Nina, a 21-year-old woman, is an example of the embodied sense of violation that harassment creates. She describes being subjected to the unwanted touch of a stranger in public transportation when still underage, and feeling that it was “really wrong.” However, she continues to explain that at the time she did not have the words to make sense of the encounter. Understanding the encounter as being “wrong” thus emerged here through embodied knowledge, despite lacking a corresponding vocabulary:

And then he put his hand on my thigh and started then sort of rubbing it, and then I started feeling that this is somehow really wrong, and this is really disgusting, and that this should not happen, but I did not sort of have a word of its own for it, that what it is like, at that age, even if we had surely talked about it at home, like that no one is allowed to touch you, but we had not verbalised in any way this kind of situation. (Woman, age 21, interview)

Acknowledging sexual harassment as a frequent and critical issue requires the ability to recognize its varied forms and manifestations—that is, being able to name various experiences of violation located at different points on the continuum of sexual and gender-based violence (see e.g., Kelly, 2012) as harassment. At other parts of her interview, Nina (as well as many other participants) describe this as a process of learning enabled by conversations with both her parents and later with her friends, in which their orientation to harassment and violence shifts as their knowledge of it accumulates. It appears that in these processes, embodied, affective knowledge of the wrongness of violations (Ahmed, 2018) increasingly attaches to discursive, socially shared knowledge. Hence there is a shift in the levels of certainty regarding wrongness, a reshaping of understanding allowed for by being capable of connecting one's experiences to socially and discursively shaped, evaluative system of thought. This process escapes a clear-cut distinction between non-knowing and discursively assisted knowing, and instead underlines their entwinement, which continues to evolve over time. Simultaneously, the sense of sexual violations’ omnipresence seems to grow. Several participants described this learning process as a recent or ongoing one, with no definite end point. Referring to a “grey area” between showing sexual interest and harassment, Lydia, a 29-year-old ciswoman, notes that a level of uncertainty remains to this day in defining each encounter:

It has been difficult for me in the past, especially when I was younger, to perhaps trust my own definition and the right to define what constitutes sexual harassment […] We have also been taught to ignore things a lot. By us, I mean maybe girls and those raised as women; we have been taught to ignore, for example, on the street if someone comes to suggest something outrageous and suggestive while coming after me. I was not able to understand before that this was sexual harassment. Even though I can walk away from this situation, and even though it's over in a moment, that is still harassment. So defining is not always easy. And then there is a certain grey area there just because we may not always have the social skills to take certain situations forward. When is flirting, for example, sexual harassment? (Ciswoman, age 29, interview)

This extract shows how the participants linked the uncertainty in recognizing and naming sexual harassment to socialization into cultural norms of tolerance and minimization of sexual harassment, which contribute to its insidiousness by attaching stealth to its omnipresence. The uncertainty in naming particular experiences as sexual harassment can be traced to the effects of hegemonic discourses that normalize sexual harassment (Gavey, 2019), the power of which the participants themselves commonly recognized. Although the participants usually described having mostly moved past such uncertainty, they did not view recognizing harassment as self-evident. Based on their seeing both the omnipresence of sexual harassment and the enduring difficulties in recognizing it, the participants frequently called for increased awareness of the issue and highlighted the importance of “talking about it” so that an increasing number of people could gain the ability to recognize and rebuke sexual harassment. In doing so, they enacted complaints against the culture of silence that they saw as continuing to surround the issue (Ahmed, 2021). This criticism was often combined with a firm stance against accepting the sense of inferiority attached to being a target of harassment, as the following response of Skyler, a 15 years old, nonbinary young person, to the question of how sexual harassment could be prevented indicates.

It needs to be talked about, and it needs to be talked about by me. That's perhaps most important. But then, it also needs to be defined clearly for young people that this is harassment, that this is not a joke, that you don’t have to tolerate this, and just saying that when you experience sexual harassment, there's no requirement that you need to, like, tolerate it. That you have not been given this kind of a role in the society that when you go out on the street, you need to be afraid. (Nonbinary, age 15, interview)

In this extract, Skyler highlights the need to assist young people in learning to recognize their right not to be harassed, as well as acquiring the ability to name their experiences as harassment. In addition to calling for adults and educators to take responsibility for facilitating such a learning process, the participants highlighted the value of social media and campaigns such as the #metoo movement in inducing this process. In the following extract, Sharon, a 21-year-old woman, describes how starting to actively discuss sexual harassment on social media triggered others to share their experiences of harassment. This, in turn, helped her grasp the commonality of sexual harassment:

Well, I would say that it [sexual harassment] is common and, well, especially when I started talking about these things on social media, many of my friends—I mean amazingly many—came to say that they have experienced these—and not just catcalling or such, even though that is horrible too, but even like rapes and all, as brutal as it can be. And then, I do feel that sexual harassment is more like a rule than an exception. (Woman, age 21, interview)

The sense of a shared understanding of sexual harassment as a serious issue and the increased attention given to it as a positive development was expressed by participants with varying gender identities. However, there was not absolute unanimity on the issue: there were also a small number of dissenting views, expressed succinctly in the online survey and mainly by participants identifying as men, that the increased attention given to sexual harassment was not a positive development (see Venäläinen & Berg, 2025). These isolated views deviated from the bulk of the views in the materials especially due to expressing concern over an assumed prevalence of false accusations against men, thereby aligning with online men's rights rhetoric articulated in opposition to movements such as #metoo (cf. Horeck et al., 2023). Nevertheless, whereas previous studies have pointed out gender differences in the ways sexual harassment is talked about (e.g., Honkatukia et al., 2023; Horeck et al., 2023), considering the overall patterns in the materials of the present study, there appeared to be more like-mindedness regarding the seriousness of the issue among the participants, with more men aligning with the emphasis on taking the matter seriously than with men's rights rhetoric. In the following extract, for instance, Garey, a 23-year-old self-identified heterosexual cisman, expresses his gratefulness for social media discussions on harassment and their capacity to raise awareness:

I only became aware of the topic a few years ago, when a friend told me about their experiences. Undeniably shocking that such a big problem is swept under the rug and normalised. […] I am grateful that the discussion is taking place. I have come across a lot of discussions, especially in Instagram, there has been a lot of discussion about it. (Heterosexual cisman, age 23, written response)

The extract exemplifies the learning process to understand the severity of sexual harassment as an issue by listening to others’ experiences of harassment, which social media is seen to facilitate. Thus, even though the emphasis on the severity of the problem is similar here to the ways sexual harassment is described by the participants (mostly women and non-binary people) who link it to their own experiences, the process of acquiring such knowledge is different. Making sense of the process described here calls for expanding the idea of experiences of injury as a (necessary) basis for understanding the seriousness of sexual violations; whereas most participants based their knowledge of the seriousness of the issue on their personal experiences, here the process appears as a vicarious one. This aligns with the observations of Hearn et al. (2023) that personal experience is not the only route to feminist knowledge, which can also be produced by men whose knowledge is based on learning by listening to others’ experiences of inequality. Certainly, the knowledge created through a vicarious process is not equivalent to the knowledge shaped by personal experiences of sexual harassment (which also a few men among the participants told about), but neither should it be assumed that learning by personal experience produces same kind of knowledge for all; rather, I suggest reading the entanglements of understandings and experiences as always-already shaped by difference and fluidity.

In sum, the key message that the participants expressed through their critical observations and recollections of the influence of sexual harassment on their and other people's everyday lives was that sexual harassment continues to shadow the lives of many, and this needs to be acknowledged better than it has been. Such knowledge was seen as requiring a learning process to name certain behaviors and experiences as harassment, in which social media and sharing experiences and views on harassment can play an important, positive role.

### The Persistence of Victim-Shaming

The second overarching understanding/experience coalesced around victim-shaming and its condemnation. Victim-shaming is frequently considered a central component in secondary victimization, which refers to traumatic experiences that are compounded upon primary victimization, such as disrespectful or belittling treatment by authorities when reporting incidents of sexual harassment and violence. This is intimately bound with victim-blaming attitudes and practices, which place the responsibility for harassment and violence on the victims themselves (Lazard, 2020). Rape myths play a key role in these dynamics; they are based on views that sexual harassment and violence happen only to women who “ask for it” by the way they dress or behave (such as consuming alcohol), and the places they frequent (such as public spaces known to be dangerous after dark) (Gavey, 2019). These beliefs and patterns were recognized and strongly criticized by the participants; indeed, many of their ideas of needed changes revolved around their prevalence. The participants recounted instances of victim-blaming both on a more abstract level, such as on social and traditional media, and in their own social encounters. In the next extract, Keira, a 19-year-old woman, describes an incident faced by her acquaintance in which the police treated the victim with disregard. In Keira's account, the telling of the incident reinforces her argument that sexual harassment is an issue that continues to be dismissed.

The police did not express any sympathy for the victim. One of my acquaintances became a target of sexual harassment when drunk. The police did not do anything about it, and this person had to continue their life as usual. They were left to suffer the trauma and the fear towards men caused by the situation. (Woman, age 19, written account)

What is notable in Keira's account is that, somewhat similarly to Garey's in the previous section, her critique is based on a vicarious process of learning the failing of social justice in how sexual harassment and its victims are treated. This again highlights the sociality and variability of the processes whereby understandings and experiences of sexual violations merge. Exceeding in a similar manner a concern for individuals and instead highlighting social patterns, other participants also pointed out that in their ideal future, “the police no longer measure the severity of a sexual crime by the length of the skirt” (15-year-old woman, written account). In addition to police and other authority figures, mental health professionals’ ability to respond to victims of harassment were frequently seen as weak, which was linked to secondary victimization. In the next extract, Aida, a 29-year-old ciswoman, recounts her experience with a sex therapist who questioned her depiction of an incident as sexual violence, insinuating that not having expressed her unwillingness forcefully enough could have been a relevant factor:

I’ve been to a sex therapist myself, who, ummm, asked me that, when I told them about nonconsensual sex, they asked me, “Well, but did you say no?” and questioned my experience of it being rape. And it was just awful that I had gone there to get help for these problems, and this was the reception. So somehow I would hope that when a young person dares to seek help that there would be an adult who really knows about these things and knows their own responsibility and knows the position of power that is inevitably there and which they cannot alter; there is a certain power dynamic, and somehow I would hope that at least professionals who work with these issues and with these young people would be really aware of the issues they are teaching. (Ciswoman, age 29, interview)

The extract exemplifies a complaint about prevalent practices and the inadequate knowledge of professionals, including a lack of awareness of the power inscribed in their professional positions. Similar to the criticism described above targeting the culture of silence, here, the reproach and awareness of the system's failures are based on the participants’ own experiences of mistreatment. Whereas in this extract, the participant appears capable of rejecting the position of a responsible party that the professional puts on offer for her, in many other descriptions of the aftermath of harassment, the participants described difficulty in doing so, in a way that closely aligns with the notion of secondary victimization. Particularly, trying to come to terms with shame was the most prevalent form of *violence work* (Vera-Gray & Kelly, 2020) that the participants described. The concept of violence work refers to active efforts on behalf of victims to mend the harm done by harassment. Reversing shame is described as requiring specific work to regain a sense of worth, and the need for such work was described as an enduring challenge that victims of harassment must face. For instance, Melanie, a 27-year-old ciswoman, described that in the wake of being harassed, “This feeling of being like dirty and being objectified kind of drags on, so you keep working on reminding yourself of your own worth.”

In many accounts, the constant risk of self-blame or even self-hate was exacerbated by victim-shaming. The ongoing need to deflect its effects also comes through in the next extract, in which Nikki, a 26-year-old ciswoman, responds to a question asking what advice she would give to someone who has experienced sexual harassment:

Mmm, just that it's not your fault. That you have done nothing that could lead to it. It's the harasser's fault, and they should not act like that. And that there may come those feelings of shame, but that you should not be left alone with them but to talk to someone about it. And always that it's not your fault, and of course that these things can evoke all kinds of emotions and that it's ok to feel them, but you should not blame yourself for it. (Ciswoman, age 26, interview)

The force of victim blame and shame and the persistent need to deflect their effect is evident in the repetition of the phrase “not your fault,” to which the participant returns after acknowledging the emergence of contradictory feelings. In a sense, she suggests the necessity of not trusting one's feelings in a conscious effort to resist victim blame. In Ahmed's (2004) terms, victim shame appears in the participants’ accounts as *sticky*, both in terms of its enduring effect on the victims themselves as well as in terms of acting as a recurring element in the process of attaching meaning to sexual harassment and the people it involves in the cultural imaginary. Put in the context of the #metoo era, characterized by increasing efforts to dispute the legitimacy of traditional victim-blaming discourses, these experiences contrast starkly with the sense of empowerment that seems to spread as a result of collective online condemnation of sexual harassment. Thus, the critical understandings of sexual harassment and experiences of shame converge in this mode of understanding/experience in a discrepant way. They can be seen as constituting a lived dilemma (Towns & Adams, 2009; see also Venäläinen & Calder-Dawe, 2024), in which beliefs and values that align with efforts to enhance gender equality and disrupt violent practices are countered by experiences of devaluation that have a powerful affective impact. This dilemma and awareness of it appeared to give specific force to the participants’ criticism and their determination to speak out against sexual harassment. Thus, similar to the understanding/experience of the omnipresence of sexual harassment, it combined with an agentic, activist orientation that most participants’ descriptions exhibited.

In sum, the key message the participants put forth when exposing and criticizing victim-shaming and blaming was that these are still prevalent and that sexual harassment victims need better support and understanding. The force of this message came from recollections of their own experiences or observations of the prevailing patterns of victim blaming and their affective force, which creates the “stickiness” of shame on both a cultural and personal level.

### Non-Action as the Prevalent Norm

This has already been talked about on social media for years, for example, the Me Too campaign and so on, but really, I feel that even though we are talking about this, nothing is being done about these problems. (Woman, age 21, interview)

As this extract exemplifies, many participants felt that “nothing is being done” about the problem of sexual harassment. Similar to the lived dilemma of continued shame described in the previous section, this notion created a sense of contradiction when considered alongside the recently intensified attention given to the topic. Non-action as the prevailing norm was the third understanding/experience identified in the analysis. Descriptions of non-action were often generated in response to a question presented to the participants about what they thought should be done to tackle the problem of sexual harassment. These responses included stating what should not be done and what should not be left undone.

Overall, the participants’ talk/texts about sexual harassment typically included various descriptions of shortcomings in prevailing efforts to intervene in sexual harassment. In highlighting the inadequacy of intervention by describing a lack of change, the participants frequently combined memories of their past events with the patterns they witness today. For instance, Jordan, a 20-year-old self-identified agender young person, writes both in past and present tense when describing the persistent lack of concern about the issue. Jane, a 26-year-old woman, in turn, describes that she never told anyone about her encounter with sexual harassment when she was in school, and through this recollection, she identifies a need for support services:

The school didn’t take it seriously enough, even though I informed the teacher about it. Harassment was not talked about enough when we were younger, and the seriousness of the issue was not emphasised. The topic is made fun of and when it is talked about, it is not talked about seriously enough. (Agender, age 20, written account)

Now that I think about my own youth, I don’t remember that I would have ever told it to any adults. I never told my parents. That maybe there should be a school curator at school or someone there to whom you can go to tell, or just somewhere you can go to talk about it and who could intervene. (Woman, age 26, interview)

The conflating of past and present in these accounts reiterates the perception of no apparent shift in the practices, and thus underlines the prevailing nature of non-action. These accounts of the failure of authorities, such as school staff, to intervene, and the lack of an easily accessible place to go and talk about one's experiences were echoed in several participants’ descriptions. This criticism led them to underline the importance of training professionals to recognize and deal with harassment in a similar fashion to that described by Aida in the previous section. Interestingly, whereas, for instance, the examples above come from participants in their 20s who talk about school contexts based on their memories from a few years back, very similar lamentation of non-action in schools also characterized younger, 15- and 16-year-old participants’ accounts. Thus, the fact that these participants’ experiences at school occurred in different time periods—for one group ongoing currently while for the other in the past—did not reveal differences in their responses. Hence, the understanding/experience of non-action, particularly in school contexts, appears to cross generations, and continues in the #metoo era in much the same fashion as before.

The participants, however, did not simply state and condemn the prevalence of non-action or inadequate action when responding to the question of tackling sexual harassment. Rather, they often provided several suggestions for change, such as strengthening sex education in schools, improving professionals’ training, and raising public awareness on platforms such as social media. In doing so, despite expressing frustration at the current state of non-action, they ultimately construed the problem of sexual harassment as solvable. This is quite a different view of sexual harassment compared to those commonly held by young women in Finland in the early 2000s, when sexual harassment was primarily seen as an unavoidable part of everyday life that one simply learns to cope with (Aaltonen, 2017). This suggests that, for these participants, sexual harassment had become a tangible issue that depends on human behavior that can and should be altered—in a word, it has become solvable. Their common mode of relating to the problem is thus also characterized by adopting an agentic position and participating in efforts to change the current state of affairs.

Furthermore, the solutions proposed by the participants were often underpinned by a multilevel understanding of a need to alter individuals’ behavior and to change cultural and societal norms that maintain notions of women's, as well as gender and sexual minorities’ inferiority, which justify and normalize sexual harassment targeting them. Some participants explicitly talked about change occurring through the gender system, such as by “exposing misogynist societal structures and fixing them” (Ciswoman, age 29, written account). In other words, their views were often informed by a systemic understanding of harassment, which enabled them to see it as part of a broader pattern of gender-based violence rooted in gender inequality (Kelly, 2012). Furthermore, the participants belonging to gender and/or sexual minorities formulated a norm-critical systemic view of sexual harassment that highlights the intertwining of gender norms and the othering of queerness in the practices that sustain and legitimize sexual harassment. Carla, a 20-year-old queer ciswoman, for instance, stressed that harassment should be prevented with education “not just involving consent principles but also eliminating biases regarding the fetishization of queer women.” Like Carla, many participants thus recognized the impact of the entwined gender- and hetero-normativities in sustaining harassment, and readily spoke out against them. In sum, then, the understandings/experiences of non-action as the prevalent norm cut across the younger and older age ranges, and were often coupled with explicit ideas of how and what should be changed. Furthermore, the participants’ calls for action were frequently underpinned by a systemic understanding of the force of cultural normative practices in sustaining harassment and violence.

## Discussion

The young people who participated in this study were highly cognisant of sexual harassment as a form of violence, and readily criticized its prevalence, its underlying normativities and gendered inequalities, and a general lack of engagement with the issue. This pattern of criticism clearly differs from the opposing pattern of minimizing the severity of sexual harassment that was identified in studies conducted in Finland in the early 2000s (Aaltonen, 2017; Venäläinen et al., 2023), and which has likewise been seen more broadly in Western countries as part of dominant discourses on sexual harassment (Gavey, 2019). Furthermore, whereas previous studies have pointed out gender differences in the ways sexual harassment is talked about (e.g., Honkatukia et al., 2023; Horeck et al., 2023), in this study there was more convergence than differences in the ways the participants, including women, non-binary people and men, emphasized the seriousness of the issue. These observations provide support for earlier studies’ cautious optimism regarding potential changes in perceptions of sexual harassment following the #metoo movement (e.g., Lazard et al., 2023). However, similar to previous findings (e.g., Skoog et al., 2023), this study affirms that sexual harassment is still very much an issue that shadows young people's everyday lives and routinely causes them harm, with significant consequences for their social relations and self-perceptions. Furthermore, the participants’ accounts indicate that the harm caused by sexual harassment continues to be exacerbated by patterns of secondary victimization and a lack of systematic practices of intervention in institutions such as schools or among welfare professionals. In sum, what this study specifically highlights alongside shifts in young people's capabilities to make sense of sexual harassment through critical lenses is the persistent shadow that sexual violations cast on young people across time periods. This largely manifests as similarities in experiences of harassment and ways it is responded to across differences in for instance age and gender identities among the participants.

This paper sought to extend previous research on young people's own ways of making sense of sexual harassment and violence (both as collective and individually experienced phenomena) in the current cultural climate by specifically attending to the relationship between experiences and understandings of sexual violations. Informed by theorization on situated knowledges and a new materialist understanding of onto-epistemological entanglements that form phenomena such as sexual harassment (Venäläinen, 2023), the analysis sought to illuminate not just experiences *or* understandings, but rather what I call understandings/experiences, a term that underscores the inseparability of these dimensions of reality. As the analysis showed, the participants themselves treated their experiences as key sources of knowledge of sexual harassment, and drew on them in formulating a critique of sexual harassment. However, attending to the relationship between these dimensions also showed variability in the processes of learning that led the participants to express critique against sexual harassment; whereas the majority of participants (especially women and nonbinary people) drew on their own experiences, they (including participants who self-identified as men) also frequently drew on vicarious experiences of witnessing sexual violations. Therefore, closely analyzing the processes of learning that connect experiences to understandings can also shed light on differences and thus variability in the ways such processes unfold.

I suggest that the notion of inseparability of experiences and understandings helps locate and give weight to the participants’ calls for action; they do not simply reiterate digitally spreading understandings but also draw on their own embodied histories of contact with the issue in formulating their proposals for solving it. By adopting this notion, research can therefore take another step toward attending to young people's lived, varied and always emerging discursive-material realities. I claim that this is a prerequisite for grasping complexity in their ways of relating to the issue of sexual harassment and violence in the contemporary era.

A new materialist understanding of onto-epistemologies, and thus seeing reality as a constantly unfolding process that manifests itself in multiple ways (Davies, 2020), calls for considering what the research and its findings themselves enable—that is, how the research participates in the unfolding of reality by illuminating certain understandings/experiences, and what kind of potential for positive change this entails. Analytically, a valuable starting point is what Davies (2016) has called *emergent listening*, which specifically attends to that which captures our attention, perhaps by diverting from our expectations, and thus “glows” (Maclure, 2013) in research materials. However, this does not mean that the analyst would be capable of stepping out of the process of interpretation, or that attempting to do so would be considered desirable. Similar to the key premise of RTA (Braun & Clarke, 2012), subjectivity (or multiple subjectivities) can be seen as a resource for interpretation, thereby integrating the researcher's experience-based knowledge into the analytical process aimed at illuminating collective patterns. As an analyst, I have in many ways approached the participants’ accounts from the outside: I do not belong to the category young people and, though no stranger to sexual harassment, unlike many of the participants I have had only limited exposure to recent social media accounts of these issues. Regardless of these differences, there was a great extent of similarity between my own experiences and understandings and my participants’ accounts, which made it relatively easy to plug all of these into the larger figurations of patriarchy. This deepened the emerging understanding of the continuities in understandings/experiences of harassment across different times and spaces. Furthermore, what also enabled the convergence of my own and my participants’ understandings/experiences in this research process were the workshops organized as a part of this study, in which the issues of sexual harassment and gender were tapped into both through discussion and creative exercises. Whereas a detailed account of the workshops falls outside the scope of this paper, it is important to recognize their role in generating spaces for mutual learning and collaborative shaping of both my and the participants’ understandings; they created new experiences of engagement with the issue for us all, which (re)-invigorated our critical orientations. I therefore view the workshops as having crucially helped to shape shared understandings/experiences related to these issues.

In the current study, steps to enhance the relevance and effectiveness of the generated knowledge with the help of collaborative research practices were also taken by seeking to hear the participants’ views on the analysis and informing the interviewees of the possibility of commenting on the initial interpretation of overarching themes in the materials. A few participants did comment and provided further support for the meaningfulness of the analysis from their perspectives. They specifically emphasized the value of attending to diverse experiences and including people who belong to gender or/and sexual minorities in the study. Even these small measures of collaboration with the participants in this study proved important in helping to align, at least partially, researcher and participant understandings, and ultimately in interfering in the formation of epistemic hierarchies between these. In conclusion, more research needs to be done *with* young people on both their everyday exposure to sexual harassment and on their views on how to enact change, to collaboratively engage in exploring ways to intervene in the patterns that maintain sexual violations. This study suggested that many young people have the desire to make a difference in society regarding these issues, and in collaboration with researchers and practitioners their possibilities to do so grow significantly.

In terms of the practical implications of this study, then, I suggest listening closely to the participants’ views on how to intervene in violence and help its victims/survivors that crystallize their understandings/experiences. These views underscore the value of increased awareness of gendered inequalities, education that cultivates respect for other people and their boundaries, and open discussion of sexual harassment that challenges its shamefulness and the culture of silence. The specific area of participants’ criticism was victim-shaming and blaming, accompanied by calls for increased sensitivity and professionals’ ability to grasp and counter secondary victimization in their encounters with victims/survivors. In addition to these practical measures, the participants emphasized the need to address the root causes of sexual harassment—the culture of violence upheld by normative understandings of gender and sexuality that maintain the idea of the inferiority of women and various minorities to justify their harassment.
